# Participant Demographic and Baseline Drinking Factors Can Predict Alcohol Use Disorder Pharmacotherapy Clinical Trial Completion and Drinking Outcomes

**DOI:** 10.1111/acer.70288

**Published:** 2026-04-09

**Authors:** Michaela Hoffman, Raymond F. Anton, Arnie Aldridge

**Affiliations:** ^1^ Department of Psychiatry and Behavioral Sciences Medical University of South Carolina Charleston South Carolina USA; ^2^ RTI International, Research Triangle Park Durham North Carolina USA

**Keywords:** alcohol use disorder, AUD treatment, clinical trials

## Abstract

**Background:**

Partially due to the complexity associated with conducting trials, there have been relatively few regulatory agency‐approved medications for the treatment of alcohol use disorder (AUD). Heterogeneity of study samples, such as participant characteristics (including variation in alcohol consumption) as well as drinking efficacy endpoints, may lead to inconsistency in results and a potential increase in Type II errors. The aim of this study is to begin to fill in the knowledge gap of optimal clinical trial design by analyzing potential predictors of outcomes.

**Methods:**

Five federally funded and publicly available multisite randomized pharmacotherapy clinical trials for the treatment of AUD using similar methodologies to assess drinking outcomes were included. Three FDA‐guided drinking efficacy outcomes were calculated for each study independently and combined: abstinence (no drinking days), no heavy drinking days, WHO 2+ risk drinking level (RDL) reduction as well as a measure of study participant completion. These outcomes were analyzed by logistic regression models including a variety of predictors within the full‐study sample as well as among only participants treated with placebo.

**Results:**

The number of days abstinent prior to randomization was a strong positive predictor of all three predefined drinking outcomes. Further analysis indicated a cutoff of less than three to five abstinent days before randomization might be optimal. For the WHO 2+ RDL endpoint, those within the “high” or “very‐high” risk categories were more likely to meet criteria for successful two or more risk level reductions than “medium” risk. Across various demographic variables, only age (older participants) was associated with better outcomes.

**Conclusions:**

These findings suggest that some important prestudy drinking characteristics (e.g., less abstinence and older age) as well as being in a higher risk drinking category should be considered for inclusion in future alcohol pharmacotherapy trials.

## Graphical abstract

Five multisite AUD pharmacotherapy trial data were merged to explore participant prestudy characteristics as outcome predictors. Pre‐randomization “days since last drink” significantly predicted three drinking outcomes. Further analyses suggested that more than 3–5 days of consecutive abstinence would reduce chances of finding a significant medication effect, secondary to increased placebo response.

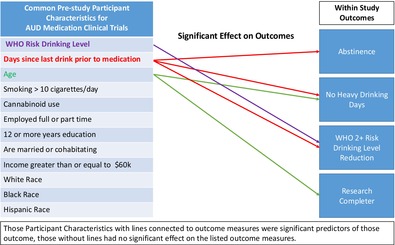

## 1 Introduction

While there have been significant increases in our understanding of the neurobiological mechanisms that underlie alcohol use disorder (AUD), only three medications are currently FDA approved for its treatment (disulfiram, naltrexone, and acamprosate). Slow and costly medication development is partially due to the limited knowledge of the most appropriate randomized clinical trial (RCT) design for AUD clinical trials. Numerous simultaneous decisions must be made before the start of an RCT that impacts both the cost and the outcomes such as sample size, number of sites, inclusion and exclusion criteria, and the length of the trial. The impact of different levels of these factors in trials of AUD populations is unknown. Additionally, there are concerns about the placebo effect in participants where considerable levels of reduction are commonly seen in the placebo‐treated samples (Litten, Castle, et al. 2013). This issue may be mitigated by modifying some of the trial design factors. Previous research has indicated that the baseline drinking levels of participants can be indicative of placebo response, where studies with high severity populations saw less placebo response than those with mild severity populations (Scherrer et al. 2021).

While traditional medicine clinical trials may have outcomes such as mortality or recovery that can be defined and measured clearly either directly or with surrogate measures, with AUD outcomes, the results depend mainly on drinking outcome summaries which rely mostly on self‐report data. Daily consumption is typically collected via a calendar‐based method called the timeline follow back (TLFB), which records daily drinking over a period of time (Sobell and Sobell 2000). The TLFB has been validated, but the daily drinking data must be summarized into aggregated distinct outcomes. The FDA has previously approved total abstinence (no drinking days) or no heavy drinking days over the total study period as acceptable outcomes for AUD RCTs. However, these have been observed to be overly restrictive definitions of success with low rates of study participants achieving these results. More recently, a drinking reduction metric, the World Health Organization risk drinking level (WHORDL; Witkiewitz et al. 2025), has been approved as an efficacious AUD clinical trial outcome by the FDA (Food and Drug Administration 2025). This novel “drinking reduction” outcome will hopefully enhance the ability to design more successful AUD RCTs, a goal on which this paper expands.

Using datasets from completed and well‐designed federally funded AUD clinical trials, this report aims to examine some potential study design factors impacting AUD trials to provide necessary information to reduce future trial error variance that might impact participatory or placebo effects and thereby improve the chances of successfully identifying an active medication effect such as reducing type II error estimates. Five multisite clinical trials for AUD are currently available through NIAAA. While each studies a different medication and some elements of their study design differ, there is considerable overlap. For this study, the five datasets have been merged across their drinking outcomes and baseline variables, providing a large dataset that will provide new insight into effects that can generalize to all AUD clinical trials.

The initial focus of these analyses is to test the predictive abilities of the study sample characteristics, especially baseline drinking levels and demographics, to examine their potential to produce placebo effects. Drinking levels and prior alcohol use could be controlled via inclusion criteria, with minimum or maximum levels/conditions proscribed. While demographics of a sample cannot be easily constrained and often should not be restricted by investigators, the information about the potential impact of demographic variables might provide guidance on sample sizes needed within and across sites to account for sample variance that are associated with positive or negative outcomes independent of study medication effects. By retrospectively modeling these factors, the aims of this paper are to begin to fill the knowledge gap of how to better plan an RCT for AUD by attending to important baseline demographic and alcohol consumption variation and reducing the impact of the placebo effect. We will first identify the significant predictors of outcomes among the full population and the placebo only sample. Then, we will look closer into those predictors that are identified as significant to identify what levels may make a practical difference in outcomes to provide guidance for future clinical trials.

## 2 Materials and Methods

Data included in this study come from five multisite clinical trials of pharmacotherapies for AUD. Data from all five are publicly available through the NIAAA website (https://www.niaaa.nih.gov/research/data‐archive‐resources/niaaa‐controlled‐datasets/). The COMBINE study (Anton et al. 2006) contains 1383 participants that were given either 1000 mg acamprosate three times per day, 100 mg naltrexone once a day, both, or a placebo. This treatment study was conducted over 16 weeks with a year of follow‐up data. This study also evaluated the medication treatments with and without a combined behavioral intervention (CBI) condition that was shown to subsequently obscure the main medication effect. Because of this, and since CBI was not included in the other studies evaluated for this report, participants who received CBI were removed from the COMBINE sample leaving 607 participants. Horizant (Falk, Ryan, et al. 2019) collected data on 346 participants given either gabapentin enacarbil 600 mg twice a day or a placebo. The study period was the longest of the included studies, at 26 weeks, including a week of titration at the beginning and a week of taper at the end. Participants in the Levetiracetam trial (Fertig et al. 2012) received 2000 mg a day Levetiracetam XR or placebo for 16 weeks, with a titration period of 4 weeks at the beginning of the study and a taper period of 2 weeks at the end. For Varenicline (Litten, Ryan, et al. 2013), participants received 1 mg varenicline tartrate twice a day or placebo for 13 weeks including a 1‐week titration period. In the Quetiapine trial (Litten et al. 2012), participants were given 400 mg quetiapine daily or placebo for 12 weeks, with 2 weeks of titration at the beginning, and a week of taper at the end. These five datasets were selected because they are freely accessible and they used similar methodology for data collection, in particular the TLFB method for the assessment of alcohol consumption at baseline and throughout the trial period. In sum, there are *N* = 1507 participants in the final harmonized dataset.

**FIGURE 1:**
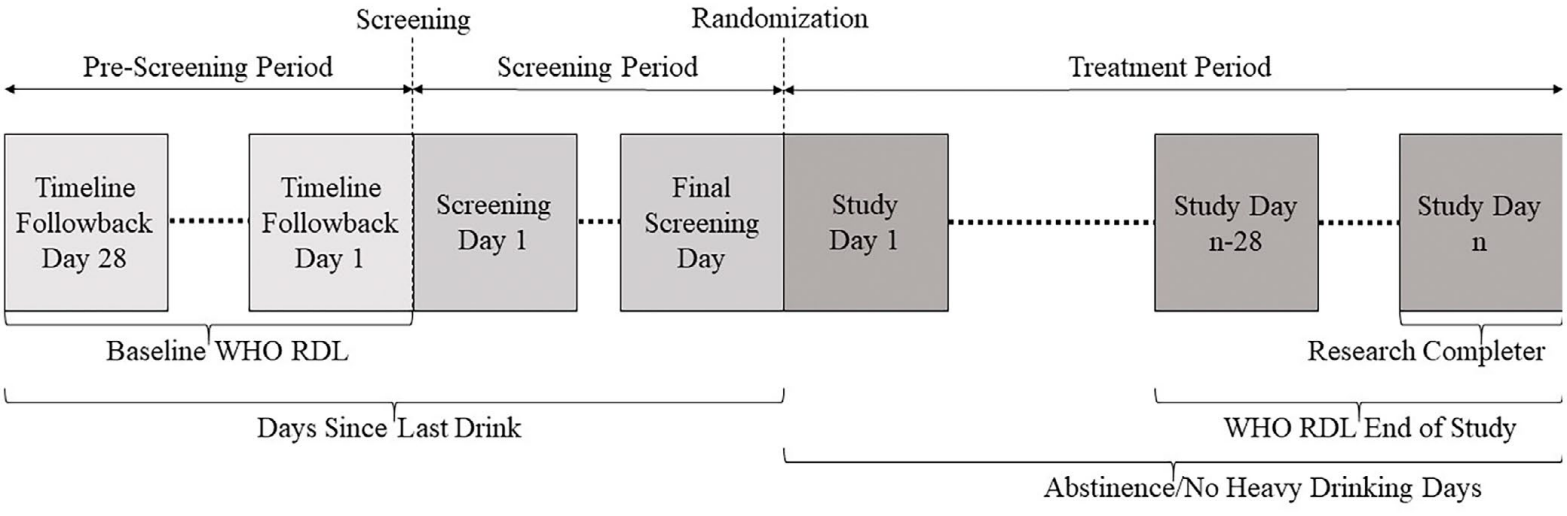
Diagram of study periods, indicating which period's timeline follow‐back (daily alcohol consumption) were used for baseline and outcome assessments. (1) “*n*” represents the length of each study. Baseline WHO RDL utilized the last month before screening. (2) Days since last drink was derived from both the pre‐screening and screening periods to determine the consecutive abstinent days immediately prior to the day of randomization. (3) Total abstinence and no heavy drinking days’ outcomes used the full treatment period. (4) The WHO RDL outcome used the last 28 days of the study (independent of study length “*n*”). (5) Research Completer status was based on the final day of the treatment period in each study.

While there is variation in the choices of measures assessed in the different studies, there are many commonalities in addition to the TLFB. Through the harmonization process, many demographic variables were collapsed with only minimal inconsistencies: gender, age, marital status, employment, income, and race. Marital status was discretized into married or cohabitating versus the remaining options (separated, divorced, widowed, never married). Employment was discretized into being employed full or part time versus the remaining options (unemployed, disabled, retired, student, military). Income was discretized into above and below $60,000 per year (income data were collected in ordinal categories), and this cutoff was selected as closest to an even split of the data. For race/ethnicity, White, Black and Hispanic categories were included. The remaining categories included too few participants to analyze: 46 mixed raced, 13 Native American, and 4 Asian/Pacific Islander. While smoking status was assessed with different scales across studies (the Form‐90 in COMBINE and Fagerström in the rest), a variable indicating whether participants smoked 10+ cigarettes per day was present in all studies. Cannabinoid (THC) use was assessed via baseline urine drug screen in all five studies providing a binary variable indicating its presence. The TLFB data for the last 28 days prior to screening were used to calculate prestudy WHO RDL, a categorical variable based on the quantity of drinks per day during the 28‐day period (see Table S1; Falk, O'Malley, et al. 2019). Since the full harmonized dataset included some participants (16 total) whose baseline WHO RDL was “low risk,” these were removed from the data, as their risk drinking over the course of the study could not attain a two‐level shift reduction. Days since last drink (DSLD) is the count of consecutive abstinent days prior to the day of randomization/first study medication dosing (as such, this includes interim days between screening and randomization). Figure 1 depicts a timeline of the study periods from which each of the outcome variables was calculated. Table 1 lists the included demographic and drinking variables as well as their rates within the included studies individually and aggregated (total) across studies.

**TABLE 1:** Baseline sample characteristics and within study drinking outcomes of the five clinical trials and final merged dataset. *p*‐values reflect an overall difference across studies using a chi‐squared test for categorical variables or ANOVA test for continuous variables (age and DSLD).

Study names	Combine		Horizant		Levetiracetam		Quetiapine		Varenicline		Total		*p*
*N*	594		346		130		221		200		1491		
	Count	%	Count	%	Count	%	Count	%	Count	%	Count	%	
Baseline characteristics													
Placebo‐treated participants	151	25.4	173	50.0	66	50.8	116	52.5	101	50.5	607	40.7	0.00
Age (mean, SD)	44.2	10.2	49.8	11.0	44.4	11.9	45.4	9.6	45.5	11.6	45.9	10.9	0.00
Sex (male)	407	68.5	225	65.0	99	76.2	176	79.6	142	71.0	1049	70.4	0.00
Married or cohabiting	270	45.5	185	53.6	52	40.0	104	47.3	90	45.0	701	47.1	0.05
12 + Years Education	543	93.5	339	98.0	120	92.3	206	93.2	190	95.0	1398	94.6	0.02
Employed full or part time	428	72.3	263	76.5	84	64.6	152	68.8	148	74.0	1075	72.3	0.08
Income ≥ $60 k	257	44.0	187	57.4	51	39.2	84	38.2	96	48.5	675	46.3	0.00
Race/ethnicity													
White	458	77.1	249	72.0	86	66.2	189	85.5	135	67.5	1117	74.9	0.00
Black	46	7.7	64	18.5	41	31.5	34	15.4	57	28.5	242	16.2	0.00
Hispanic	71	12.0	33	9.8	2	1.5	6	2.7	4	2.0	116	7.8	0.00
Cannabinoid use	70	11.8	33	9.5	14	10.8	35	16.1	22	11.0	174	11.7	0.22
Smoke ≥ 10 cigarettes per day	198	33.4	42	12.2	26	20.0	84	38.4	38	19.1	388	26.2	0.00
Days since last drink, DSLD (mean, SD)	7.8	5.2	3.5	1.5	0.6	1.8	1.8	4.2	0.2	0.6	4.27	4.9	0.00
Baseline WHO risk drinking level^a^													
Medium risk	44	7.4	18	5.2	2	1.5	2	0.9	0	—	66	4.4	0.00
High risk	132	22.2	99	28.6	6	4.6	10	4.5	22	11.0	269	18.0	
Very high risk	418	70.4	229	66.2	122	93.9	209	94.6	178	89.0	1156	77.5	
Within‐study drinking outcomes													
Abstinent	111	18.7	12	3.5	7	5.4	18	8.1	3	1.5	151	10.1	0.00
No heavy drinking day	177	29.8	23	6.7	11	8.5	32	14.5	11	5.5	254	17.0	0.00
WHO 2+ RDL reduction	410	69.0	149	43.1	59	45.4	116	52.5	91	45.5	825	55.3	0.00
Research completer	547	92.1	279	80.6	108	83.1	181	81.9	171	85.5	1286	86.3	0.00

^a^ For the WHO risk drinking levels, low risk is defined as up to 40 g per day for males and 20 for females. Medium risk is 40–60 g per day for males and 20–40 for females. High risk is 60–100 g per day for males and 40–60 for females. Very high risk is > 100 g per day for males or > 60 g per day for females.

In this study, four binary outcome variables, all based on TLFB daily drinking data, are included. While the studies differed in total length, the TLFB data from the last 28 days of the study period were used to calculate endpoint WHO risk drinking level (RDL). Participants who reduced their drinking by two or more risk levels, compared to their risk level at baseline, were considered successes (WHO 2+ RDL reduction). Dropouts and missing data were recoded to “no change” for WHO 2+ RDL reduction (negative outcome). Two other discrete drinking variables, participants with (1) total abstinence or (2) no heavy drinking days, were calculated based on the TLFB data for the entire treatment period for each study, both using “worst case scenario imputation,” assuming drop out days were a return to drinking/heavy drinking. The final outcome variable (research completer) was coded “1” if the participant reported data for the final TLFB study day and “0” if the data were missing a proxy for study completion. The counts of these study outcomes are broken down by study and in aggregate as provided in Table 1. Most of the baseline and outcome variable rates did differ by study, and for this reason, each individual study was coded and included as a covariate for the subsequent models predicting outcomes.

Logistic regression models predicting the binary (total abstinence. no heavy drinking day) endpoint drinking variables were fit with baseline drinking and demographic variables, controlling for study (as above). These were conducted on the full harmonized dataset (*n* = 1491), including a medication versus placebo predictor to control for medication effects. As a supplementary analysis, the logistic models were conducted separately on as subset of participants treated with placebo only (*n* = 610). *p*‐value significance was Bonferroni corrected to account for the four different outcomes being modeled and set to *p* < 0.05/4 = 0.0125.

Exploratory analyses of the variables found to be significant predictors were conducted, such as receiver operating curve (ROC) analysis with Youden's Index (Youden 1950) to define informative and implementable cutoffs for continuous variables where appropriate. Youden's index allows the researcher to evaluate the balance of sensitivity and specificity at potential cutoff values for the given outcome and will achieve a maximum value at the ideal cutoff point. We hypothesized that sample characteristic variables will have some predictive value for study drinking outcomes and study completion, independent of medication and unique study effects.

## 3 Results

Initial results of the logistic models for the full sample can be found in Table 2. The models indicated that DSLD prior to randomization was a significant predictor (*p* < 0.001) for all three drinking outcome variables. On average, participants with more abstinent days before randomization did better in the study (more with abstinence, no heavy drinking days, and WHO 2+ RDL reduction). Means and standard deviations of baseline DSLD within each study efficacy drinking outcome group are detailed in Table 3.

**TABLE 2:** Study baseline demographic and drinking variables associated with various within‐study drinking outcomes in study participants. Positive regression coefficients (*β*) indicate that an increase in the variable or the presence of the characteristics indicates a more positive outcome.

Baseline predictor variables	Drinking outcomes							
	Abstinent		No Heavy		WHO 2+		Research completer	
			Drinking days		RDL reduction			
	*β*	*p*	*β*	*p*	*β*	*p*	*β*	*p*
Constant	−4.20	0.00	−2.06	0.01	−1.58	0.01	2.40	0.00
Study		0.02		**0.00**		**0.00**		**0.00**
Placebo versus active medication	−0.09	0.67	−0.12	0.48	−0.22	0.07	−0.17	0.30
Baseline WHO RDL		0.74		0.08		**0.00**		0.72
High risk	−0.12	0.81	−0.52	0.14	1.86	**0.00**	0.16	0.73
Very high risk	0.09	0.83	−0.71	0.03	1.82	**0.00**	−0.03	0.94
Days since last drink	0.17	**0.00**	0.13	**0.00**	0.08	**0.00**	−0.04	0.10
Age	0.02	0.02	0.04	**0.00**	0.01	0.15	0.03	**0.00**
Smoke > 10 cigarettes per day	−0.17	0.44	−0.24	0.19	−0.17	0.21	0.20	0.33
Cannabinoid use	0.20	0.52	0.23	0.37	−0.28	0.13	0.17	0.52
Sex (male)	0.15	0.51	0.07	0.68	0.25	0.05	0.10	0.59
Employed full or part time	0.17	0.46	0.13	0.48	−0.09	0.52	−0.27	0.17
12+ years education	−0.07	0.87	−0.31	0.36	−0.14	0.61	0.09	0.81
Married or cohabiting	0.35	0.12	0.36	0.04	−0.02	0.88	−0.12	0.49
Income ≥ $60 k	−0.37	0.12	−0.11	0.55	0.22	0.12	0.00	0.99
White	0.00	1.00	−0.87	0.03	−0.23	0.44	−0.99	0.02
Black	−0.19	0.74	−0.24	0.59	0.65	0.05	−0.44	0.36
Hispanic	−0.15	0.80	−1.00	0.04	−0.39	0.23	−0.80	0.04

*Note:* The medium risk category is used as the reference/control group for the *Baseline WHO Category* variable. Regression weights and *p*‐values are from the logistic model. *p*‐values are bold where significant, using *α* = 0.05 cutoff corrected for familywise error rate across four tests gives a threshold of *p*=0.0125.

**TABLE 3:** Number of days since last drink (DSLD) at time of randomization (mean and SD) at baseline within various drinking outcome criteria categories. DSLD was a significant predictor in the logistic regression models for abstinent, no heavy drinking days, and WHO 2+ RDL reduction. DSLD had less effect on the overall study completion.

Outcome	Model *p* ^a^	DSLD		*t*	*p*
		Mean	SD		
WHO 2+ RDL reduction	< 0.001	5.3	5.4	9.52	0.00
No change		3.0	3.7		
Abstinent	< 0.001	9.9	6.4	11.72	0.00
Not abstinent		3.6	4.2		
No heavy drinking days	< 0.001	8.2	6.2	11.71	0.00
1+ heavy drinking day		3.5	4.1		
Research completer	0.1	4.4	4.9	2.03	0.04
Non‐completer		3.6	4.6		

^a^ From Table 2 logistic regression model.

Baseline WHO RDL category was predictive of the WHO2+ RDL reduction, such that a medium risk was significantly different from both high‐ and very high‐risk categories. Age was the only demographic predictor of no heavy drinking days and of study completion where participants who had no heavy drinking days were older on average (47.7 years vs. 45.5 years) and completers were older (46.1 years vs. 44.0 years). Results for the placebo‐only sample were the same in patterns of significance and can be found in Table S2.

### 3.1 Days Since Last Drink Prior to Randomization

**FIGURE 2:**
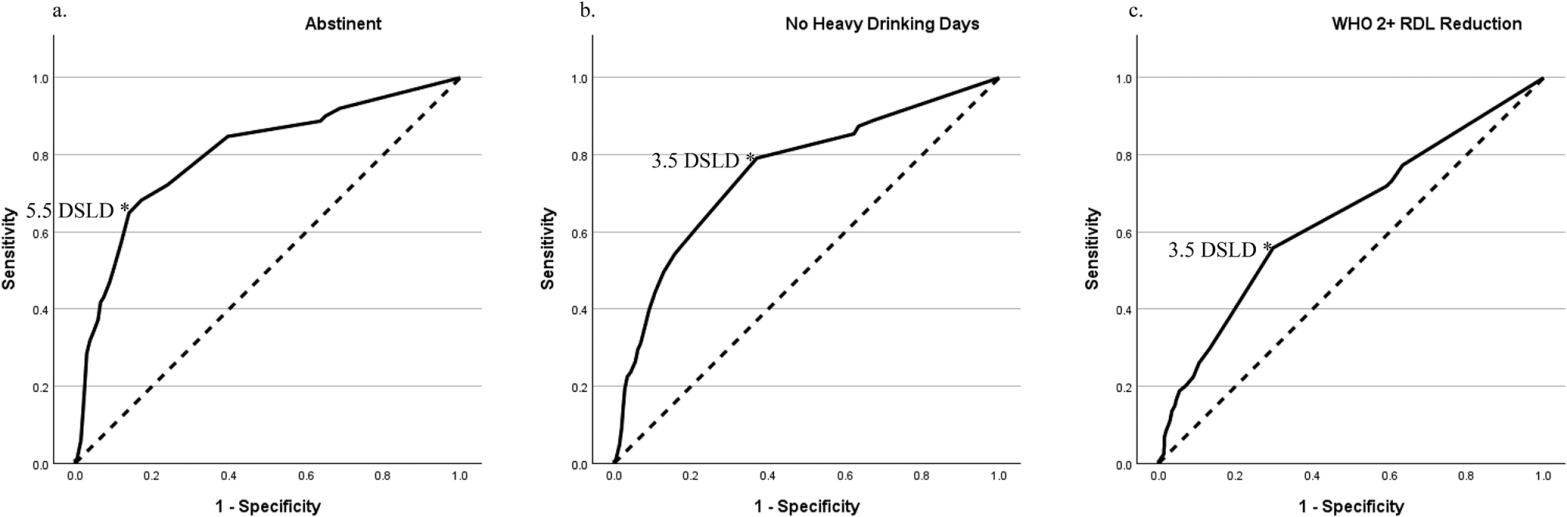
Receiver operating curves for day since last drink prior to randomization (DSLD) predicting within study drinking outcomes: Abstinence (a), no heavy drinking days (b), and WHO 2+ RDL reduction (c). The dashed line is the reference line that represents a random classifier. The more area above the reference line indicates improved classification—more true positives (sensitivity) and fewer false positives (specificity). Asterisks indicate where Youden's index is maximized. Values for sensitivity and specificity can be found in Table S2.

Since DSLD (or days of continuous abstinence prior to randomization/first dosing) was a strong predictor of all drinking outcomes even among placebo only participants, it was investigated further. Ideally, a cutoff or maximum number of abstinent days prior to randomization might be predetermined and specified to reduce a placebo effect and increase the contrast with active medication effects. A receiver operating curve (ROC curve) was created for DSLD for each of the three drinking outcomes for which it was significant. These ROC curves can be seen in Figure 2, and the values and Youden's indices can be found in Table S3. Youden's index was maximized at abstinence 3.5 days or less for the WHO 2+ RDL reduction and no heavy drinking days endpoints, and at 5.5 days or less for abstinence endpoint in both the full dataset and the placebo only dataset (Table S4). Further evaluating a cutoff of 3.5 days or less of abstinence prior to randomization in the homogenized dataset, as presented in Table 4, participants who had 4 or more days of abstinence prior to randomization had significantly better outcomes across all the drinking outcome measures (*p* < 0.001 for each) than those with three or fewer. This was true across the full study samples as well as in the placebo‐only group alone, with the exception of research completion for the placebo‐only participants (*p* = 0.033).

**TABLE 4:** Counts and percentages of participants who successfully attained each study drinking outcome broken down by a days since last drink cutoff of 3.5 days, as suggested by the ROC analyses.

Days since last drink	Participants that met criteria				Chi^2^
	0–3 days		4 or more days		
	*n*	%	*n*	%	*p*
Within‐study drinking outcome					
Abstinent	23	2.8	128	19.4	0.00*
No heavy drinking days	53	6.4	201	30.5	0.00*
WHO 2+ RDL reduction	364	43.8	461	70.0	0.00*
Research completer	688	82.7	598	90.7	0.00*
Total	832		659		

*Note:* with a correction for four tests, a *p*‐value cutoff of 0.0125 is used to denote significance, marked with an asterisk.

### 3.2 Baseline WHO Risk Drinking Level as A Predictor of Successful Treatment

Although the baseline WHO‐RDL drinking variable only significantly predicted the WHO 2+ RDL drinking reduction outcome variable, it was further analyzed on the other drinking outcome variables as well. Table 5 contains the counts and percentages of participants within each baseline WHO RDL that met criteria for the other drinking outcomes success categories. When the outcome is a reduction in the WHO RDLs, the higher risk categories are more likely to attain a reduction. Medium risk drinkers are the least likely to meet a two‐level RDL reduction, likely due to the fact that they must be fully abstinent at the end of the study to achieve success status. For the other outcome drinking measures, total abstinence, and no heavy drinking days, baseline medium‐risk drinkers are the most successful, though not significantly.

**TABLE 5:** Number of participants and percentages for the within‐study drinking outcome variables by baseline WHO risk drinking levels. Baseline WHO RDL was a significant predictor of WHO 2+ level risk reduction (see Table 2), specifically the medium risk group was less likely to achieve a 2+ level risk reduction than the high‐risk or very high‐risk groups (*p* < 0.001 for both). Although not significant, the medium risk group was the most likely to achieve abstinence and no heavy drinking days.

Baseline WHO RDL	Abstinent	No heavy drinking days	WHO 2+ RDL reduction	Research completer
	*n* (%)	*n* (%)	*n* (%)	*n* (%)
Medium risk (*n* = 66)	9 (13.6)	21 (31.8)	18 (27.3)	59 (89.4)
High risk (*n* = 269)	24 (8.9)	51 (19.0)	164 (61.0)	238 (88.5)
Very high risk (*n* = 1156)	118 (10.2)	182 (15.7)	643 (55.6)	989 (85.6)

*Note:* Participants may be included in multiple categories simultaneously (e.g., all abstinent participants had no heavy drinking day).

## 4 Discussion

These findings highlight some factors relating to sample characteristics that can impact AUD clinical trial drinking and completion variability. The count of abstinent days prior to randomization was a consistently strong predictor of study drinking level outcomes across medication and placebo groups. Participants able to maintain abstinence on their own might be more prone to the independent main effects of study participation and/or placebo (pill taking) effects. Reducing or controlling for abstinence duration before randomization, for instance, might be beneficial in reducing these effects and thereby improving the chances of finding a true medication effect, that is, reducing type II error. The follow‐up ROC analysis indicated that less than 3–5 days of abstinence prior to randomization/first dosing might be optimal to reduce the placebo effect. This, of course, must be balanced by study assessment requirements requiring abstinence for accuracy as well as other clinical factors like the possibility of alcohol withdrawal complicating treatment and medication adverse effect assessments post randomization. The important message, however, is that the more abstinence days prior to study medication initiation is likely to influence the detection of study drug effectiveness leading to increased type II error rates.

The WHO 2+ RDL reduction is somewhat dependent on the level of drinking at baseline determined over a period of time (e.g., 28 days prior to screening) and also aggregates alcohol consumption over drinking and abstinence days. Furthermore, it appears that, in participants at a “medium risk” level prestudy, there are several important observations: (1) these participants are much less likely to meet the reduction in 2+ risk level reduction criteria since to obtain that level they would have to be completely abstinent in the month or so at the end of the study and (2) they might be more likely to meet the overall criteria for total abstinence and having no heavy drinking day. These observations need further consideration as one or both could lead to increased placebo effects. All of these issues should be taken into consideration when setting drinking requirements for study inclusion.

Understanding the effect of demographic variables, while not always controllable in a study, can inform the sample size analyses when estimating medication effect size probabilities. Age was a predictor of no heaving drinking days and study retention. It is likely that older individuals have accumulated alcohol‐related consequences over time and might have more to lose by not modifying their alcohol consumption which in turn leads to greater study compliance and motivation to change. It has generally been observed that AUD clinical trial participants are of middle age, even though they might have met AUD criteria many years prior to seeking treatment. However, its impact on treatment success is worthy of further consideration as to better uniquely serve individuals of different ages and AUD histories. Further consideration of how various participant demographic variables, like participant age, might interact with other variables (such as pre‐randomization drinking levels or risk categories) and might provide additional information on the amount of variance to expect in an AUD clinical trial.

Biological markers of alcohol consumption (e.g., PEth, CDT, and EtG) are being used more broadly in clinical practice as well as in clinical trials. The ultimate hope is that these lab tests combined with self‐reported drinking will enhance validity and reduce variability of recorded alcohol consumption during AUD clinical trials thereby reducing type 2 error. They will likely play an important role in the future of trials; however, their use in the five studies included here was not universal or standardized in collection, leading to the inability to validly utilize the data available. This suggests that more standardization and potential FDA guidance on the use of these available biomarkers is considered in future AUD clinical trials.

## 5 Limitations

This study brings together five datasets to produce a relatively large sample of AUD randomized placebo‐controlled clinical trial data that were collected in a standard fashion (e.g., using TLFB calendar drinking data collection). Despite the many methodological similarities, other factors likely need to be considered. For example, each had a somewhat different trial length, used different active medications, and had varying numbers of clinical trial sites. These are all variables that cannot be fully evaluated within the constraints of these limited number of studies and sample sizes.

The generalizability of clinical trial results to a broader patient population remains a challenge. In our study, as noted in the methods, several racial categories were excluded from our analyses. Those demographic groups had representation of 3% or less, making analyses of questionable validity and increasing estimate error, potentially leading to inaccurate interpretation.

The work presented here attempted to use available standardized clinical trial data sponsored by NIAAA and coordinated under clinical research organization supervision (somewhat of a gold standard for regulatory type clinical trials). In all medical clinical trials, including those for AUD, there are specific criteria for inclusion and exclusion (many directed by FDA guidelines and many approved by the FDA a priori). In the case of AUD, the field is in evolution attempting to define “best practices” for regulatory clinical trials. This paper attempts to assist in that evolutionary process. Part of that process is to better define what AUD clinical trial participant populations are best to study to enhance clinical trial efficacy. Admittedly, as with most clinical trials, inclusion and exclusion criteria likely will not encompass the totality of the population seeking treatment. Ultimately, it will be the judgment of regulatory bodies and clinicians as to whether the population studies reflect the clinical population for which a medication is deemed effective. This judgment might call for further study (such as in potential Phase 4 FDA trials). For instance, one question (out of many) that might arise is whether a treatment‐seeking AUD individual must stop drinking for a few days prior to the initiation of treatment or whether being abstinent for too long would not indicate the need for medication treatment. This study cannot go beyond the data presented but does lay the groundwork for further study and analysis going forward.

## 6 Future Directions

Future work should include efforts to add more and larger AUD clinical trial datasets to the pool of currently available study data. Attaining additional clinical trials for these analyses will allow a more comprehensive evaluation of the impact of other important study design characteristics, as suggested above, on study outcome variability. Building on this work, these and other variables related to sample composition and study design will be subsequently utilized to elucidate optimal AUD trial design and form the basis for future potential artificial intelligence (AI) analyses. Continuing work in this area will include adding further variables, as those that can be harmonized across the datasets already identified. The overarching goal of this work is to better understand what factors influence a placebo response to maximize the ability of detecting a true medication effect. This is most important in a heterogeneous population disorder like AUD where variability in participant demographic and drinking histories as well as other factors (comorbid disorders, genetic germ line, acquired, or epigenetic differences) add substantial variability to clinical trial outcomes. As such, the work presented here is a “first step” in a longer process of evaluation and understanding of these issues.

## Funding

This manuscript is the result of work supported by the National Institute on Alcohol Abuse and Alcoholism P50 AA010761 and the Medical University of South Carolina's Department of Psychiatry Chairman's Research Development Fund Pilot Grant Program.

## Conflicts of Interest

The authors declare no conflicts of interest.

## Supporting information


**Table S1:** The WHO risk drinking levels (WHO RDL) defined for males and females. Drinking levels are based on daily averages from 28‐day timeline followback drinking periods in grams of alcohol, converted from drinks per day.
**Table S2:** Regression weights and *p*‐values from the logistic model using the placebo‐treated sample to predict drinking outcomes with baseline demographics and drinking measures. Variables marked with an asterisk were significant, corrected for familywise error rate across four tests.
**Table S3:** Results from the ROC curve analysis for the full data (placebo and active subjects). Area under the curve (AUC) statistics are presented, reflecting the ability of the predictor (days since last drink) to predict the outcomes, where an AUC of 0.5 is equivalent to random guessing. The coordinates of the ROC curve are also presented along with the Youden's index at each cutoff. Maximizing Youden's index represents the optimal cutoff value for the predictor.
**Table S4:** Results from the ROC curve analysis for the placebo only data. Area under the curve (AUC) statistics are presented, reflecting the ability of the predictor (days since last drink) to predict the outcomes, where an AUC of 0.5 is equivalent to random guessing. The coordinates of the ROC curve are also presented along with the Youden's index at each cutoff. Maximizing Youden's index represents the optimal cutoff value for the predictor.

## Data Availability

The data that support the findings of this study are available from NIAAA. Restrictions apply to the availability of these data, which were used under license for this study. Data are available from https://www.niaaa.nih.gov/research/data‐archive‐resources/niaaa‐controlled‐datasets with the permission of NIAAA.
