# Supplementary material for: Participant Demographic and Baseline Drinking Factors Can Predict Alcohol Use Disorder Pharmacotherapy Clinical Trial Completion and Drinking Outcomes
Source: Alcohol Clin Exp Res (Hoboken). 2026 Apr 9;50(4):e70288. doi: 10.1111/acer.70288 (PMC13066717; doi:10.1111/acer.70288)
Supplement: Supplementary file 1 — Table S1: The WHO risk drinking levels (WHO RDL) defined for males and females. Drinking levels are based on daily averages from 28‐day timeline followback drinking periods in grams of alcohol, converted from drinks per day. Table S2: Regression weights and p‐values from the logistic model using the placebo‐treated sample to predict drinking outcomes with baseline demographics and drinking measures. Variables marked with an asterisk were significant, corrected for familywise error rate across four tests. Table S3: Results from the ROC curve analysis for the full data (placebo and active subjects). Area under the curve (AUC) statistics are presented, reflecting the ability of the predictor (days since last drink) to predict the outcomes, where an AUC of 0.5 is equivalent to random guessing. The coordinates of the ROC curve are also presented along with the Youden's index at each cutoff. Maximizing Youden's index represents the optimal cutoff value for the predictor. Table S4: Results from the ROC curve analysis for the placebo only data. Area under the curve (AUC) statistics are presented, reflecting the ability of the predictor (days since last drink) to predict the outcomes, where an AUC of 0.5 is equivalent to random guessing. The coordinates of the ROC curve are also presented along with the Youden's index at each cutoff. Maximizing Youden's index represents the optimal cutoff value for the predictor. [file ACER-50-0-s001.docx]

**Supplementary Table 1.** WHO Risk Drinking Levels (WHO RDL) defined for males and females. Drinking levels are based on daily averages from 28-day timeline followback drinking periods in grams of alcohol, converted from drinks per day.

| **WHO Risk Drinking Level** | | **Males** | **Females** |
| --- | --- | --- | --- |
| 0 | Abstinent | 0 | 0 |
| 1 | Low | 1-40 g (<2.9 drinks) | 1-20 g (<1.4 drinks) |
| 2 | Moderate | 41-60 g (2.9-4.3 drinks) | 20-40 g (1.4-2.9 drinks) |
| 3 | High | 61-100 g (4.3-7.1 drinks) | 40-60 g (2.9-4.3 drinks) |
| 4 | Very High | ≥101 g (>7.1 drinks) | ≥61 g (>4.3 drinks) |

Note: Drinks refer to Standard Alcohol Drinks when measured as 1.5 ounces of Spirits, 5 ounces of wine, or 12 ounces of beer. These amounts are equivalent to 14 grams of alcohol.

Supplementary Table 2. Regression weights and p-values from the logistic model using the **placebo treated sample** to predict drinking outcomes with baseline demographics and drinking measures. Variables are marked with an asterisk were significant, corrected for familywise error rate across four tests.

| **Baseline Predictor Variables** | **Abstinent** | | **No Heavy** | | **WHO 2+** | | **Completer** | |
| --- | --- | --- | --- | --- | --- | --- | --- | --- |
|  |  |  | **Drinking Days** | | **RDL Reduction** | |  |  |
|  | β | p | β | p | β | p | β | p |
| Constant | -4.30 | 0.02 | -3.00 | 0.03 | -2.15 | 0.05 | 2.22 | 0.11 |
| Study |  | 0.05 |  | **0.00** |  | 0.13 |  | **0.01** |
| Baseline WHO RDL |  | 0.57 |  | 0.34 |  | **0.00** |  | 0.73 |
| Medium Risk | -0.95 | 0.34 | -0.72 | 0.33 | 2.12 | **0.00** | -0.36 | 0.66 |
| High Risk | -0.46 | 0.60 | -0.98 | 0.16 | 2.36 | 0.00 | -0.53 | 0.50 |
| Days since last drink | 0.20 | **0.00** | 0.18 | **0.00** | 0.12 | **0.00** | -0.04 | 0.34 |
| Age | 0.03 | 0.11 | 0.05 | **0.00** | 0.01 | 0.30 | 0.03 | **0.01** |
| Smoke > 10 cigarettes per day | 0.09 | 0.83 | -0.26 | 0.46 | -0.26 | 0.27 | 0.31 | 0.33 |
| Cannabinoid Use | 0.46 | 0.42 | 0.11 | 0.83 | -0.19 | 0.55 | 0.84 | 0.08 |
| Sex (male) | -0.53 | 0.18 | -0.35 | 0.27 | 0.41 | 0.04 | 0.06 | 0.83 |
| Employed Full or Part Time | 0.13 | 0.77 | 0.27 | 0.44 | -0.37 | 0.09 | -0.22 | 0.47 |
| 12+ Years Education | 0.02 | 0.98 | -0.17 | 0.77 | -0.54 | 0.23 | -0.01 | 0.98 |
| Married or Cohabiting | 0.30 | 0.47 | 0.52 | 0.11 | 0.14 | 0.49 | 0.09 | 0.74 |
| Income ≥ $60k | -0.71 | 0.12 | -0.48 | 0.16 | 0.09 | 0.69 | 0.04 | 0.88 |
| White | 0.16 | 0.86 | -1.04 | 0.14 | -0.33 | 0.48 | -0.76 | 0.26 |
| Black | 0.74 | 0.48 | 0.26 | 0.74 | 0.94 | 0.08 | 0.41 | 0.60 |
| Hispanic | 0.59 | 0.53 | -0.59 | 0.49 | -0.27 | 0.57 | -0.35 | 0.52 |

Supplementary Table 3. Results from the ROC curve analysis for the full data (placebo and active subjects. Area under the curve (AUC) statistics are presented, reflecting the ability of the predictor (days since last drink) to predict the outcomes, where an AUC of .5 is equivalent to random guessing. The coordinates of the ROC curve are also presented along with the Youden’s index at each cutoff. Maximizing Youden’s index represents the optimal cutoff value for the predictor.

|  | Abstinence | | | No Heavy Drinking Days | | | WHO 2+ RDL Reduction | | |
| --- | --- | --- | --- | --- | --- | --- | --- | --- | --- |
| AUC: |  | 0.800 |  |  | 0.755 |  |  | 0.639 |  |
| DSLD Cutoff | Sensitivity | 1 - Specificity | Youden's Index | Sensitivity | 1 - Specificity | Youden's Index | Sensitivity | 1 - Specificity | Youden's Index |
| -1.0 | 1.000 | 1.000 | 0.000 | 1.000 | 1.000 | 0.000 | 1.000 | 1.000 | 0.000 |
| 0.5 | 0.921 | 0.687 | 0.233 | 0.890 | 0.674 | 0.216 | 0.773 | 0.634 | 0.140 |
| 1.5 | 0.901 | 0.650 | 0.251 | 0.874 | 0.635 | 0.239 | 0.732 | 0.605 | 0.127 |
| 2.5 | 0.887 | 0.637 | 0.251 | 0.854 | 0.622 | 0.232 | 0.719 | 0.592 | 0.127 |
| 3.5 | 0.848 | 0.396 | 0.451 | 0.791 | 0.370 | 0.421 | 0.559 | 0.297 | 0.261 |
| 4.5 | 0.722 | 0.240 | 0.482 | 0.618 | 0.221 | 0.397 | 0.375 | 0.182 | 0.193 |
| 5.5 | 0.682 | 0.172 | 0.510 | 0.543 | 0.158 | 0.386 | 0.297 | 0.132 | 0.165 |
| 6.5 | 0.649 | 0.140 | 0.509 | 0.496 | 0.129 | 0.368 | 0.261 | 0.105 | 0.156 |
| 7.5 | 0.570 | 0.119 | 0.451 | 0.445 | 0.107 | 0.338 | 0.224 | 0.090 | 0.134 |
| 8.5 | 0.510 | 0.101 | 0.408 | 0.398 | 0.091 | 0.307 | 0.201 | 0.071 | 0.131 |
| 9.5 | 0.470 | 0.090 | 0.381 | 0.358 | 0.081 | 0.277 | 0.188 | 0.054 | 0.134 |
| 10.5 | 0.430 | 0.074 | 0.357 | 0.311 | 0.069 | 0.242 | 0.162 | 0.045 | 0.117 |
| 11.5 | 0.417 | 0.066 | 0.352 | 0.295 | 0.061 | 0.234 | 0.149 | 0.042 | 0.107 |
| 12.5 | 0.371 | 0.059 | 0.312 | 0.264 | 0.055 | 0.209 | 0.136 | 0.035 | 0.101 |
| 13.5 | 0.338 | 0.046 | 0.291 | 0.236 | 0.043 | 0.193 | 0.113 | 0.030 | 0.083 |
| 14.5 | 0.318 | 0.038 | 0.280 | 0.224 | 0.034 | 0.190 | 0.099 | 0.026 | 0.074 |
| 15.5 | 0.285 | 0.030 | 0.255 | 0.193 | 0.027 | 0.165 | 0.085 | 0.020 | 0.065 |
| 16.5 | 0.212 | 0.025 | 0.187 | 0.146 | 0.023 | 0.122 | 0.068 | 0.015 | 0.053 |
| 17.5 | 0.132 | 0.020 | 0.112 | 0.091 | 0.019 | 0.071 | 0.045 | 0.015 | 0.030 |
| 18.5 | 0.093 | 0.017 | 0.076 | 0.067 | 0.016 | 0.051 | 0.034 | 0.014 | 0.020 |
| 19.5 | 0.060 | 0.015 | 0.045 | 0.047 | 0.014 | 0.034 | 0.024 | 0.014 | 0.011 |
| 20.5 | 0.013 | 0.005 | 0.008 | 0.012 | 0.005 | 0.007 | 0.008 | 0.003 | 0.005 |
| 22.0 | 0.007 | 0.002 | 0.004 | 0.004 | 0.002 | 0.002 | 0.004 | 0.002 | 0.002 |
| 24.5 | 0.007 | 0.001 | 0.005 | 0.004 | 0.002 | 0.002 | 0.004 | 0.000 | 0.004 |
| 26.5 | 0.007 | 0.001 | 0.006 | 0.004 | 0.001 | 0.003 | 0.002 | 0.000 | 0.002 |
| 28.0 | 0.007 | 0.000 | 0.007 | 0.004 | 0.000 | 0.004 | 0.001 | 0.000 | 0.001 |
| 30.0 | 0.000 | 0.000 | 0.000 | 0.000 | 0.000 | 0.000 | 0.000 | 0.000 | 0.000 |

Supplementary Table 4. Results from the ROC curve analysis for the placebo only data. Area under the curve (AUC) statistics are presented, reflecting the ability of the predictor (days since last drink) to predict the outcomes, where an AUC of .5 is equivalent to random guessing. The coordinates of the ROC curve are also presented along with the Youden’s index at each cutoff. Maximizing Youden’s index represents the optimal cutoff value for the predictor.

|  | Abstinence | | | No Heavy Drinking Days | | | WHO 2+ RDL Reduction | | |
| --- | --- | --- | --- | --- | --- | --- | --- | --- | --- |
|  |  | 0.762 |  |  | 0.731 |  |  | 0.604 |  |
| DSLD Cutoff | Sensitivity | 1 - Specificity | Youden's Index | Sensitivity | 1 - Specificity | Youden's Index | Sensitivity | 1 - Specificity | Youden's Index |
| -1.0 | 1.000 | 1.000 | 0.000 | 1.000 | 1.000 | 0.000 | 1.000 | 1.000 | 0.000 |
| 0.5 | 0.822 | 0.609 | 0.214 | 0.779 | 0.602 | 0.177 | 0.676 | 0.576 | 0.099 |
| 1.5 | 0.756 | 0.571 | 0.184 | 0.740 | 0.562 | 0.178 | 0.625 | 0.548 | 0.077 |
| 2.5 | 0.756 | 0.550 | 0.206 | 0.740 | 0.540 | 0.201 | 0.604 | 0.529 | 0.075 |
| 3.5 | 0.733 | 0.281 | 0.452 | 0.701 | 0.258 | 0.443 | 0.427 | 0.210 | 0.216 |
| 4.5 | 0.644 | 0.146 | 0.499 | 0.545 | 0.130 | 0.415 | 0.270 | 0.102 | 0.168 |
| 5.5 | 0.600 | 0.100 | 0.500 | 0.494 | 0.085 | 0.409 | 0.212 | 0.067 | 0.145 |
| 6.5 | 0.533 | 0.078 | 0.455 | 0.429 | 0.066 | 0.363 | 0.177 | 0.051 | 0.127 |
| 7.5 | 0.422 | 0.068 | 0.355 | 0.351 | 0.057 | 0.294 | 0.147 | 0.045 | 0.102 |
| 8.5 | 0.400 | 0.060 | 0.340 | 0.312 | 0.053 | 0.259 | 0.133 | 0.041 | 0.092 |
| 9.5 | 0.400 | 0.057 | 0.343 | 0.312 | 0.049 | 0.263 | 0.130 | 0.038 | 0.091 |
| 10.5 | 0.356 | 0.048 | 0.308 | 0.260 | 0.043 | 0.216 | 0.113 | 0.032 | 0.081 |
| 11.5 | 0.356 | 0.043 | 0.313 | 0.247 | 0.040 | 0.207 | 0.109 | 0.025 | 0.084 |
| 12.5 | 0.333 | 0.041 | 0.292 | 0.234 | 0.038 | 0.196 | 0.106 | 0.022 | 0.084 |
| 13.5 | 0.311 | 0.030 | 0.281 | 0.208 | 0.028 | 0.179 | 0.089 | 0.016 | 0.073 |
| 14.5 | 0.289 | 0.021 | 0.268 | 0.195 | 0.019 | 0.176 | 0.072 | 0.013 | 0.059 |
| 15.5 | 0.289 | 0.011 | 0.278 | 0.169 | 0.011 | 0.158 | 0.058 | 0.006 | 0.052 |
| 16.5 | 0.200 | 0.007 | 0.193 | 0.117 | 0.008 | 0.109 | 0.041 | 0.003 | 0.038 |
| 17.5 | 0.111 | 0.005 | 0.106 | 0.065 | 0.006 | 0.059 | 0.024 | 0.003 | 0.021 |
| 18.5 | 0.067 | 0.005 | 0.061 | 0.039 | 0.006 | 0.033 | 0.017 | 0.003 | 0.014 |
| 19.5 | 0.044 | 0.002 | 0.043 | 0.026 | 0.002 | 0.024 | 0.007 | 0.003 | 0.004 |
| 20.5 | 0.044 | 0.000 | 0.044 | 0.026 | 0.000 | 0.026 | 0.007 | 0.000 | 0.007 |
| 25.0 | 0.022 | 0.000 | 0.022 | 0.013 | 0.000 | 0.013 | 0.003 | 0.000 | 0.003 |
| 30.0 | 0.000 | 0.000 | 0.000 | 0.000 | 0.000 | 0.000 | 0.000 | 0.000 | 0.000 |
